# Informal caregiving burden and perceived social support in an acute stroke care facility

**DOI:** 10.1186/s12955-018-0885-z

**Published:** 2018-04-05

**Authors:** Christopher Olusanjo Akosile, Tosin Olamilekan Banjo, Emmanuel Chiebuka Okoye, Peter Olanrewaju Ibikunle, Adesola Christiana Odole

**Affiliations:** 1 Medical Rehabilitation Department, Faculty of Health Sciences and Technology, College of Health Sciences, Nnamdi Azikiwe University, Nnewi Campus, Anambra State Nigeria; 2 Department of Physiotherapy, University of Uyo Teaching Hospital, Uyo, Akwa-Ibom State Nigeria; 3 Department of Physiotherapy, College of Medicine, University of Ibadan and University College Hospital, Ibadan, Oyo State Nigeria

**Keywords:** Stroke, Stroke survivors, Informal caregivers, Burden, Social support, Acute care, Nigeria

## Abstract

**Background:**

Providing informal caregiving in the acute in-patient and post-hospital discharge phases places enormous burden on the caregivers who often require some form of social support. However, it appears there are few published studies about informal caregiving in the acute in-patient phase of individuals with stroke particularly in poor-resource countries. This study was designed to evaluate the prevalence of caregiving burden and its association with patient and caregiver-related variables and also level of perceived social support in a sample of informal caregivers of stroke survivors at an acute stroke-care facility in Nigeria.

**Methods:**

Ethical approval was sought and obtained. Fifty-six (21 males, 35 females) consecutively recruited informal caregivers of stroke survivors at the medical ward of a tertiary health facility in South-Southern Nigeria participated in this cross-sectional survey. Participants’ level of care-giving strain/burden and perceived social support were assessed using the Caregiver Strain Index and the Multidimensional Scale of Perceived Social Support respectively. Caregivers’ and stroke survivors’ socio-demographics were also obtained. Data was analysed using frequency count and percentages, independent t-test, analysis of variance (ANOVA) and partial correlation at α =0.05.

**Results:**

The prevalence of care-giving burden among caregivers is 96.7% with a high level of strain while 17.9% perceived social support as low. No significant association was found between caregiver burden and any of the caregiver- or survivor-related socio-demographics aside primary level education. Only the family domain of the Multidimensional Scale of Perceived Social Support was significantly correlated with burden (*r* = − 0.295).

**Conclusion:**

Informal care-giving burden was highly prevalent in this acute stroke caregiver sample and about one in every five of these caregivers rated social support low. This is a single center study. Healthcare managers and professionals in acute care facilities should device strategies to minimize caregiver burden and these may include family education and involvement.

## Background

The caregiving role is often associated with experience of burden or strain by the informal caregivers who are often family members and this is particularly more evident among those caring for patients with disabling conditions like stroke [1–3]. Stroke, one of the leading causes of long-term disability, is often sudden, leaving the individual and the family to deal with confounding emotions and realities [3–8]. The early phase is often characterized by hospitalization and the presence of family members during hospitalization as seen in some cultures is regarded as being fundamental for surviving stroke [9, 10]. However the informal caregivers of stroke patients in the acute phase seem to have increased burden that bothered on the immediate health status and medical interventions required for the recipients [10, 11]. They report not knowing what to ask, impact of the situation, limited communication, use of unfamiliar medical terms by health professionals and hospital environment issues as challenges [2, 12, 13].

Higher levels of burden among stroke caregivers in a poor resource country compared to those from the western world have been previously reported [14]. Harsh economic conditions and poor institutional support alongside other factors were suggested as probable reasons for the higher burden in caregivers from lower resource countries [14, 15]. Most of the studies on informal caregiving in low resource settings were conducted among caregivers of stroke survivors undergoing out-patient rehabilitation whose level of burden may be different from those providing acute phase in-patient care. The need to study caregivers’ burden and needs across different settings and across the care continuum have been previously highlighted [16, 17].

Studies on caregiver burden in the acute phase of stroke from developed economies are rather rare, probably because in some of these countries, the patients are more likely to have adequate number of healthcare providers providing care every hour of the day till the point of discharge. The cost of care for some patients and in some countries is also borne by the health system [2, 18]. The situation is however different in the developing countries. Providing informal care for in-patients particularly in public hospitals in poor-resource countries could be worrisome and dreadful. Over-burdened healthcare professionals tend to shift some of their own responsibilities to the informal caregivers who are already faced with the previously highlighted challenges and still expect them to deliver effectively.

Social support has been identified as one of the resources that may help reduce the strain of caregiving [19–21]. Caregivers reportedly experience a higher level of burden when they have lower levels of social support [22] and respite provided by family and friends in terms of physical, emotional and financial assistance may buffer the negative effects of caregiving [23–25] For the acute phase caregiver, the social support from family and friends may be in form of hospital visits and assistance with some of the additional responsibilities fostered on caregivers by the healthcare providers, and with payment of some of the bills and sharing some of the emotional concerns of the caregivers.

## Methods

This study was aimed at investigating the level of burden and social support being experienced by informal caregivers of stroke survivors in the acute care centres and survivors- and caregivers-related socio-demographic variables associated with the constructs at an acute stroke care facility in Nigeria. The study protocol was approved by the Ethical Committee of University of Uyo Teaching Hospital (Reference Number: UUTH/AD/S/96/VOL.XII/114) and individual participant gave written and verbal consent after due explanation of the study’s procedure. Data was collected from all the available stroke survivors and their caregivers, thus giving a response rate of 100%. This was a cross-sectional survey of 56 (21 males, 35 females) informal caregivers of stroke survivors who were consecutively recruited from the medical ward of a tertiary health facility in South-Southern Nigeria. Each caregiver was more than 18 years old and was identified by self and nurses in the medical ward as the primary caregiver for the stroke survivor. The primary caregiver was defined as the person (family or non-family member) spending the most time in providing daily care for the stroke survivor or the person taking on the main caregiving tasks [26, 27] Caregiver Strain Index (CSI) is a 13-item questionnaire that has at least one item for each of the following major domains: Employment, Financial, Physical, Social and Time. Positive responses to seven or more items on the index indicate a greater level of strain and presence of burden [28]. Each participant was ranked as either burdened or not. The CSI has been reported to have a high internal consistency (α = 0.86).

The Multidimensional Scale of Perceived Social Support (MSPSS) - a 12-item instrument designed to assess the perception of social support adequacy from the sources of family, friend and significant others [29] was used to assess social support among participating caregivers. The participants were required to rate their perception on a 7-point Likert-type scale and the individual participant’s score is the sum total of the individual item scores. It ranges from 12 to 84. Higher scores (69–84) reflect higher perceived social support, moderate scores (49–68) indicate moderate perceived social support while lower scores (12–48) indicate low perceived social support. The instrument has been reported to have good internal consistency with Cronbach’s alpha (0.90), parallel form reliability (0.94) and test re-test reliability (0.76) [30, 31]. The questionnaires were researcher-administered to the caregivers in order to enhance clarity and also improve the response rate. The data collection for the present study took place over a period of 9 months (from January to September, 2014).

Data was analysed using Statistical Package for the Social Sciences (SPSS) version 20. The socio-demographic and clinical variables (age, gender, educational status, employment status, marital status, level and side of weakness, co-morbidity, residence, relationships, and whether caregiving was shared or not) were summarised using frequency counts and percentages. Independent T test and one-way analysis of variance were used to determine the influence of caregiver- and survivor-related variables on the burden scores of caregivers. Partial correlation analysis was used to determine the relationship between caregiver burden scores and each of total and domain social support scores while controlling for age, gender, educational status and occupational status of caregivers and stroke survivors; relationship of caregivers with survivors; whether the caregivers and survivors live together or not (residence); and whether caregiving responsibility was being shared or not. Alpha level was set at 0.05.

## Results

Caregivers (mean age = 28.2 **±** 9.5 years) in this study were majorly female (67.9%), children of the stroke survivor (60.7%), has a post-secondary level education (67.8%) and were either students or trade apprentices (51.8%). The stroke survivors (mean age = 57.2 **±** 13.7 years) were also majorly females (62.5%), having at least secondary level education (57.2%) and were either self-employed or working in public or private corporations (76.8%) and sharing the same accommodation with their caregivers (69.6%) prior to the stroke onset (Tables 1 and 2).

**Table 1:** Socio-Demographic Profiles of the Stroke Survivors

Variable	Category	Frequency (Percentage)
Gender	Male	21 (37.50)
	Female	35 (62.50)
Side affected	Right	28 (50.00)
	Left	28 (50.00)
Educational status	Non-formal	4 (7.10)
	Primary	20 (35.70)
	Secondary	16 (28.60)
	Tertiary	10 (17.90)
	Post-graduate	5 (8.90)
Level of weakness	Hemiparesis	42 (75.00)
	Hemiplegia	14 (25.00)
Occupation	Retired	12 (21.40)
	Selfemployed	28 (50.00)
	C/PS	15 (26.80)
	Student	1 (1.80)
Marital status	Single	7(12.50)
	Married	41(73.20)
	Widowed	8(14.30)
Co-morbidity	Nil	2(3.60)
	Hypertension	37(66.10)
	DM	3(5.60)
	HTN + DM	10(17.90)
	Others	4(7.14)

Key: *HTN* Hypertension, *Others* Osteoarthritis of the knee, eye problem, *DM* Diabetes Mellitus

*= Significant at *p* > 0.05

C/PS = Works in Public or private organization

**Table 2:** Socio-Demographic Profiles of Informal Stroke Caregivers

Variable	Category	n (%)
Gender	Male	18(32.10)
	Female	38(67.90)
Type	Sole	18(32.10)
	Shared	38(67.90)
Relationship	Partner	6(10.70)
	Son/daughter	34(60.70)
	EFM	14(25.00)
	Friend	2(3.60)
Residence	Same	39(69.60)
	Different	17(30.40)
Educational status	Non-formal	0(0.00)
	Primary	4(7.10)
	Secondary	14(25.00)
	Tertiary	32(57.10)
	Post-graduate	6(10.70)
Occupation	S/A	29(51.80)
	Self -employed	10(17.90)
	P/CS	16(28.60)
	Applicant	1(1.80)
Co-morbidity	Nil	54(96.40)
	Hypertension	1(1.80)
	DM	0(0.001)
	Others	1(1.80)

Key: *EFM* Extended family member, *Others* Osteoarthritis of the knee, eye problem, *DM* Diabetes Mellitus, *P/CS* Works in Public or private organization, *S/A* Student or Apprentice

Mean CSI and MSPSS scores were 9.3 **±** 2.0 and 60.2 ± 17.0 respectively. Nearly all the participants (96.7%) reported being highly burdened and 10 (17.9%) and 34 (60.7%) reported available social support as low and moderate respectively. There was significant influence of caregivers’ educational status on the CSI scores (Table 3). Bonferroni post-hoc analysis revealed this difference CSI score to exist between caregivers with post-graduate and primary levels education. No other caregiver- and survivor-related variables (age, gender, educational and occupational status, relationship and pre-stroke living arrangement of caregiver with survivor) had significant influence on the CSI score (Table 4).

**Table 3:** Independent t- test and Analysis of variance (ANOVA) showing the influence of participants’ socio-demographic variables on the caregiver burden scores

Variables	Class	Mean ± Standard Deviation	t/F	p
Gender of Survivors	Male	9.67 ± 2.01	0.99	0.33
	Female	9.11 ± 2.04		
Survivors’ Age (years)	1–39	9.67 ± 1.86	0.83	0.44
	40–64	9.03 ± 2.13		
	≥65	9.76 ± 1.89		
Caregivers’ Age(years)	1–39	9.22 ± 2.01	−1.08	0.28
	40–64	10.17 ± 2.14		
Gender of Caregivers	Male	8.83 ± 1.58	−1.25	0.22
	Female	9.55 ± 2.19		
Number of Caregivers	1	8.47 ± 1.88	2.42	0.10
	2–4	9.56 ± 2.02		
	5	11.00 ± 1.41		
Caregivers’ Marital status	Single	9.71 ± 1.70	2.09	0.13
	Married	9.51 ± 1.89		
	Widowed	8.00 ± 2.67		
Caregivers’ Education status	Primary	11.50 ± 1.00	3.33	0.03*
	Secondary	9.57 ± 1.87		
	Tertiary	9.25 ± 1.74		
	Postgraduate	7.67 ± 3.08		
Survivors’ Occupational Status	Retired	9.42 ± 2.84	0.13	0.94
	Self-employed	9.39 ± 1.89		
	public/civil service	9.07 ± 1.67		
	Student	10.00		
Caregivers’ Occupational Status	Student	9.55 ± 1.70	0.98	0.41
	Self-employed	8.90 ± 3.21		
	public/civil service	9.00 ± 1.63		
	Applicant	12.00		
Caregivers’ Relationship With Survivors	Spouse	10.17 ± 1.94	0.96	0.42
	Child	9.21 ± 2.17		
	OFM	9.50 ± 1.74		
	Friends	7.50 ± 0.71		
Residence	Same	9.41 ± 1.82	0.64	0.53
	Different	9.06 ± 2.49		

KEY: *OFM* other family members

*=Significant at *p* < 0.05

**Table 4:** Partial Correlation Showing the Relationship between Social Support and Caregivers’ Burden Score

Variables	r	p
CSI vs Family social Support	−0.351*	0.018
CSI vs Friend	0.091	0.552
CSI vs Significant others	−0.081	0.598
CSI vs Total Social Support	−0.124	0.418

Controlled variables:

Age of survivors and caregivers

Gender of survivors and caregivers

Educational status of survivors and caregivers

Occupational status of survivors and caregivers

Relationship caregivers with survivors

Residence of the caregivers and survivors

Whether caregiving was shared or not

Significant inverse correlation was found between caregivers’ CSI scores and only the family domain of the social support scale (*r* = − 0.30, *p* < 0.05). No correlation could be established with the other domains and overall MSPSS score.

## Discussion

The prevalence of burden and level of perceived social support among individuals providing informal care to stroke survivors in an acute care medical facility in Uyo, a city in Southern Nigeria was investigated in this study. Majority of the caregivers in the present study were children of the stroke survivors. This is similar to previous local reports [7, 14, 15]. This is not surprising considering the fact that Nigerian children have cultural and moral obligations to take care of their parents during old age or period of ill health. Hence, the responsibility of caring for ailing parents always falls on their grown-up children. It is also rather rare to see men who have grown-up children serving as main informal caregivers at in-patient facilities. Since there are more female survivors in the studied sample, it is more likely that the spouses only visit while the children stay with their mothers. Higher percentages of the stroke survivors being females may be a reflection of what obtains at the facility during the particular data collection period and not what is generally obtainable and usually reported in literature [32]. It has been reported that more males than females suffer stroke [32]. Majority of survivors living with their caregivers may also be attributed to a socio-cultural practice whereby women particularly widows tend to live with their children to assist in raising their grandchildren. It will thus not be out of place for these children to be primary caregivers at their periods of in-patient care..The higher proportion of female caregivers was however not unexpected, and is similar to the results of some previous local studies [7, 14, 15]. In Nigeria and Africa as a whole, caregiving roles are usually considered as women’s work [7].

The prevalence of burden at 96.7% is very high. Caregivers’ burden had often been evaluated post-hospital discharge while the patient is still undergoing out-patient rehabilitation or within the community to which they are discharged [2, 33]. Previous local studies [14, 15] had reported burden to be highly prevalent (83.5%) among caregivers of survivors in out-patient rehabilitation, and that the prevalence values from their studies were higher than what obtained in other climes. It would seem that aside environmental and cultural influences that may be associated with caregivers’ burden, providing informal caregiving to a patient in an acute care setting may be perceived as being more burdensome compared to the post-discharge period.

There was significant influence of caregivers’ educational status on the CSI scores with caregivers who attained postgraduate education having lower burden than those who attained only primary education. Higher educational attainment may logically correlate with chances of acquiring knowledge on stress coping strategies with resultant decrease in perceived burden. The study was unable to establish any significant influence of survivor- and caregiver-related variable on the caregivers’ burden score. Previous studies have however found age, caregivers and patients’ gender, relationship to survivor, educational attainment, employment status of caregiver to be associated with caregivers’ burden [15, 34, 35]. The present study however supports the findings of no significant relationship between caregivers’ burden and survivors’ and caregivers’ ages and gender reported by McCullagh et al. [36]. The similarity in findings between the McCullagh et al’s study and the present study may be due to the fact that caregivers in both studies were predominantly females and children of the stroke survivors.

About 20% of caregivers studied ranked their perceived social support as low though a majority of participants perceived social support as moderate with higher scores coming from the family and significant others domains. Yu et al. [27] had reported stroke caregivers in their study as perceiving insufficient social support, especially from friends and other members of their social network. Similar to this study finding, they reported the support the caregivers perceived as coming mainly from family members. This may not be surprising as family members and others in the social network such as members of the same religious or social fellowships, colleagues at the workplace or market stalls and neighbours at places of residents often visit individuals who are hospitalized as a socio-cultural duty bearing gifts in cash and kind. However, expectations from friends and significant others are not often so high and a one-time visit to the health facility often suffices and is greatly appreciated. The picture may be different for family members as they may be expected to share or partake in the burden of care along with the primary caregivers.

Participants’ scores in the family social support domain of the MSPSS was the only one among other domains and overall MSPSS scores that had a significant inverse correlation with CSI scores. This seems to buttress the earlier assumption of higher expectations from family members. Previous authors using different measures of burden and social support had also found an inverse relationship between the constructs [37]. It has been suggested that availability of other family members as support resources may lessen the burden or strain experienced by the primary caregiver [38]. However, Cameron et al. [17] reported that caregivers of stroke survivors benefitted from receiving support from health care professionals, family, friends and care-giving peers. In their study, health care professionals provided caregivers with information, instrumental, training and appraisal support; caregiving peers rendered information (on practical guidance for caring in the home) and emotional support; family and friends provided emotional and instrumental support which included home preparation; help around the home; provision of food and assistance with care provision. Providing the support in the acute phase of stroke is to be encouraged so as to prevent unwanted consequences of negative first caregiving experience. These might include the same person not wanting to continue with caregiving, adverse health effects, particularly the mental health, poor likelihood of engaging in preventive health measures, and also the eventual institutionalization of care-recipient [13, 14, 32, 38, 39]. One-half of all caregivers reportedly have at least one chronic condition [30, 40]. This study did not investigate whether these chronic conditions were adverse health effects resulting from their caregiving roles. We reason however that the caregiving period may be too short to have precipitated the conditions but also that the conditions may be worsened in the presence of caregiving burden.

The study has certain limitations which ought to be acknowledged. The burden of providing informal care for stroke survivors in the early and acute care phase in a tertiary hospital only was evaluated and so the findings may not fully represent the experience of caregivers in either primary or secondary care or private facilities. However, empirical evidence suggests that caregivers’ experiences may not be so different across the various levels of care except in private health facilities catering solely for the very high income class. The study was also conducted among stroke caregivers in a single tertiary facility based on the fact that a focus group discussion conducted prior to the study revealed similar characteristics in terms of in-patient care among most of the tertiary health facilities in our environment. The sample size of the present study may be considered to be small; hence the result of the present study may be interpreted with caution. Future studies in this area may consider using more robust sample size.

## Conclusions

The study found a rather high prevalence of burden in a stroke caregiver sample who majorly reported moderate social support from family, friends and significant others. No significant association was found between the patient socio-demographics and burden. Participants’ scores on the family domain of the social support scale was the only domain with a significant correlation with their scores on the burden scale. The results of our study indicate that strengthening the family support for informal caregivers may be an important strategy to reduce caregiver burden.

## Abbreviations

CSI: Caregiver Strain Index
MSPSS: Multidimensional Scale of Perceived Social Support
